# Loss of BOK Has a Minor Impact on Acetaminophen Overdose-Induced Liver Damage in Mice

**DOI:** 10.3390/ijms22063281

**Published:** 2021-03-23

**Authors:** Samara Naim, Yuniel Fernandez-Marrero, Simone de Brot, Daniel Bachmann, Thomas Kaufmann

**Affiliations:** 1 Institute of Pharmacology, University of Bern, Inselspital, INO-F, 3010 Bern, Switzerland; samara.naim@pki.unibe.ch (S.N.); yuniel04@gmail.com (Y.F.-M.); daniel.bachmann@pki.unibe.ch (D.B.); 2 Sunnybrook Health Sciences Centre, University of Toronto, 2075 Bayview Ave, Toronto, ON M4N 3M5, Canada; 3 COMPATH, Institute of Animal Pathology, University of Bern, Laenggassstrasse 122, CH-3012 Bern, Switzerland; simone.debrot@vetsuisse.unibe.ch

**Keywords:** BOK, BCL-2 family, acetaminophen, ER stress, UPR, apoptosis, necrosis

## Abstract

Acetaminophen (APAP) is one of the most commonly used analgesic and anti-pyretic drugs, and APAP intoxication is one of the main reasons for liver transplantation following liver failure in the Western world. While APAP poisoning ultimately leads to liver necrosis, various programmed cell death modalities have been implicated, including ER stress-triggered apoptosis. The BCL-2 family member BOK (BCL-2-related ovarian killer) has been described to modulate the unfolded protein response and to promote chemical-induced liver injury. We therefore investigated the impact of the loss of BOK following APAP overdosing in mice. Surprisingly, we observed sex-dependent differences in the activation of the unfolded protein response (UPR) in both wildtype (WT) and *Bok*^-/-^ mice, with increased activation of JNK in females compared with males. Loss of BOK led to a decrease in JNK activation and a reduced percentage of centrilobular necrosis in both sexes after APAP treatment; however, this protection was more pronounced in *Bok^-/-^* females. Nevertheless, serum ALT and AST levels of *Bok^-/-^* and WT mice were comparable, indicating that there was no major difference in the overall outcome of liver injury. We conclude that after APAP overdosing, loss of BOK affects initiating signaling steps linked to ER stress, but has a more minor impact on the outcome of liver necrosis. Furthermore, we observed sex-dependent differences that might be worthwhile to investigate.

## 1. Introduction

*Para*-(Acetylamino)phenol (acetaminophen, paracetamol, herein referred to as APAP) is one of the most commonly used antipyretic and analgesic drugs in the world [1,2]. It is safe to use within its rather narrow therapeutic window; however, an overdose can lead to severe hepatic injury and liver failure [1,3]. As an over-the-counter obtainable drug, excessive self-medication—intentional (due to suicide attempts) or unintentional (by the use of combination products containing APAP)—is the main cause of APAP-intoxication and accounts for ~50% of all acute liver failure (ALF) cases in the Western world [1,2,3,4]. The metabolism of APAP takes place mainly in the liver, with a lesser contribution of the kidney and intestine [5]. While the main portion of APAP is readily conjugated with glucuronic acid or sulphate and excreted with the urine, only a small percentage (~10%) is metabolized by cytochrome P450 (predominantly CYP2E1 and CYP1A2), leading to the formation of the highly reactive intermediate *N*-acetyl-*p*-benzoquinone imine (NAPQI) [2,4,6,7]. Under physiological conditions, NAPQI is rapidly conjugated to glutathione (GSH) and secreted in the urine as cysteine and mercapturic acid conjugates [6]. However, if GSH stores are depleted, a situation encountered upon APAP overdosing, unconjugated NAPQI binds and reacts with cellular, preferentially mitochondrial proteins, leading to mitochondrial dysfunction and cell death [6]. Importantly, it has been described that female mice are less susceptible to APAP hepatotoxicity compared to male mice [8,9], due to an accelerated GSH restoration, which allows the scavenging of more NAPQI from the cell [8].

So far, the only approved antidote for APAP intoxication is N-acetylcysteine (NAC), a precursor for GSH, which is able to bind and scavenge excessive NAPQI [1,2]. NAC has to be administered within the first 8 h of overdosing, as its efficacy significantly diminishes thereafter [3]. Due to the short therapeutic time window of NAC and the adverse effects that have been observed after prolonged treatments, more insight into cell death pathways that are activated in response to APAP overdose is needed to develop new treatment options, especially for late-presenting patients [2].

Although it is well established that APAP overdose ultimately leads to liver necrosis [2,3,10], various programmed cell death modalities have been implicated during its course. For instance, ferroptosis was reported to be activated and to aggravate the severity of liver damage [11] and its inhibition by ferrostatin-1 was associated with a decrease in hepatocyte cell death in vitro and in vivo [11,12]. Necroptosis, a RIPK1/3 and MLKL-dependent programmed cell death with necrotic outcome, is also described to be activated following APAP intoxication. However, despite induction of RIPK1/3 and MLKL, the involvement of these genes in APAP hepatotoxicity is controversially discussed (reviewed in [13]). Similarly, the contribution of apoptotic cell death to the pathophysiology of APAP-induced hepatotoxicity and the roles of several BCL-2 family members in modulating disease severity is controversially discussed (reviewed in [14]). Simultaneous downregulation of anti-apoptotic BCL-2 and upregulation of pro-apoptotic BAX after APAP treatment was correlated with increased caspase-3 cleavage, apoptotic cell death and increased hepatotoxicity [15,16,17], while an overexpression of human BCL-2 in transgenic mice unexpectedly showed enhanced hepatotoxicity [18]. Genetic deletion or silencing of the pro-apoptotic BH3-only proteins BIM, BID and PUMA were all reported to partially protect from APAP-induced liver damage [19,20,21].

Mitochondria play a crucial role in the development of APAP-induced liver damage. APAP overdose-induced ROS triggers a prolonged opening of the mitochondrial membrane permeability transition pore, which results in mitochondrial membrane potential depolarization, a drop in energy (ATP) production and, eventually, necrosis [6,22]. In addition to mitochondria, ER function and integrity are also heavily affected by APAP overdose. In fact, the transcription factor GADD153 (CHOP) is upregulated upon APAP treatment in renal tubular cells and mouse liver [23,24]. CHOP, in turn, mediates the transcriptional induction of BIM, which eventually leads to the initiation of apoptosis [25]. The induction of ER stress and subsequent activation of the unfolded protein response (UPR), is an important mechanism in the development of APAP hepatotoxicity [26,27] and *Chop^-/-^* mice were found to be protected from liver damage following APAP overdose [28]. Several studies have shown that ER stress can induce non-apoptotic programmed cell death, namely necroptosis, which may be relevant given the necrotic outcome of APAP intoxication [29,30,31]. Mechanistically, the connection from ER stress to necroptosis is poorly understood, but may involve ligand-independent activation of TNF-R1 [29] and may be facilitated in the context of ischemia/reperfusion [30,31].

BCL-2-related ovarian killer (BOK) is a member of the BCL-2 protein family with the highest structural similarity to pro-apoptotic BAX/BAK, and multiple studies have implicated BOK in pro-apoptotic processes [32,33,34,35]. Nevertheless, BOK fulfils other functions that are not, or not directly, associated with apoptosis, such as the regulation of cellular proliferation, autophagy induction and modulation of the UPR (reviewed in [36]). A role of BOK in the latter is supported by the findings that the majority of BOK is localized at the ER-membrane [37,38,39]. In the mouse liver, BOK was shown to mediate diethylnitrosamine (DEN)-induced hepatocellular apoptosis [40]. That study showed that DEN induces BOK levels in a partially JNK-dependent manner, and that compared with WT controls, livers from treated *Bok^-/-^* mice had reduced induction of CHOP, BIM and PUMA, resulting in decreased apoptosis. Given the previously reported roles of BOK in acute liver damage and in modulating UPR responses, we addressed in this study how the loss of BOK affects liver damage in male and female mice upon APAP overdosing.

Surprisingly, we found sex-dependent differences in the activation of the UPR following ER stress, and a decrease in JNK activation in *Bok^-/-^* compared to WT mice. However, whereas the loss of BOK decreased lobular necrosis in females (and to a smaller extent also in males) based on pathohistological assessment, no significant difference in overall liver damage, as assessed by ALT and AST serum levels, was detected between the genotypes.

## 2. Results

### 2.1. Expression of CYP2E1 Is Increased at the mRNA but Not the Protein Level in Bok^-/-^ Livers

**Figure 1:**
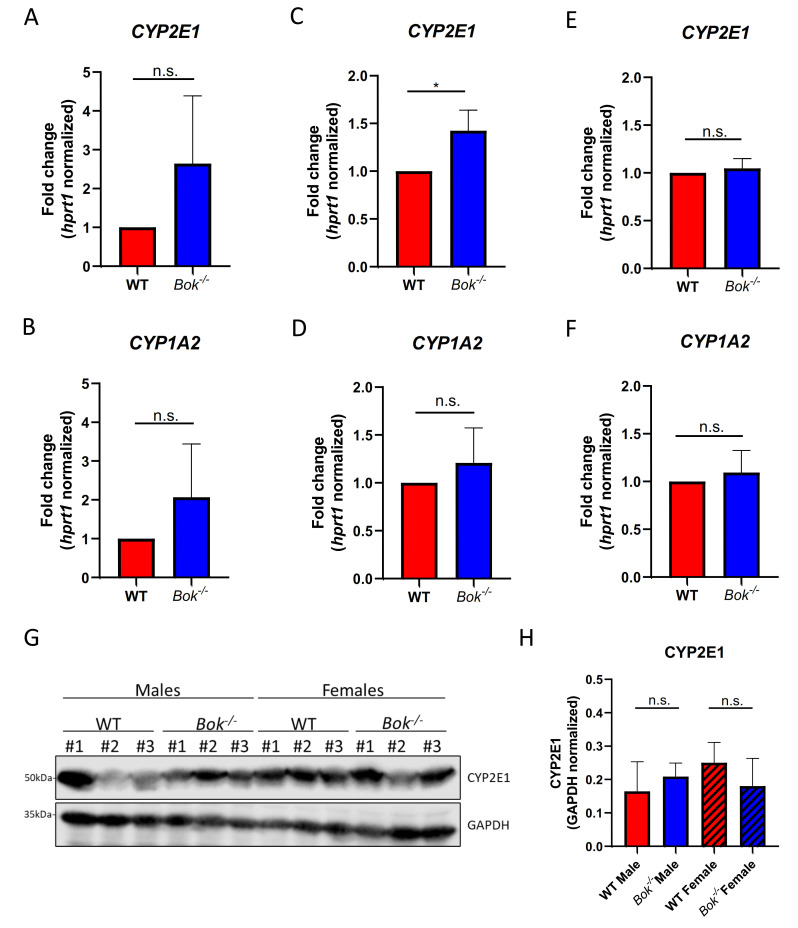
CYP2E1 mRNA but not protein expression is increased in *Bok^-/-^* males at baseline. qPCR analysis revealed increased expression of CYP2E1 and CYP1A2 in primary hepatocytes derived from *Bok^-/-^* male mice compared with wildtype (WT) (**A**,**B**). Expression of CYP2E1, but not CYP2A1 was significantly increased in whole liver lysates derived from *Bok^-/-^* males compared with WT (**C**,**D**). In whole liver lysates of female WT and *Bok^-/-^* mice CYP2E1 (**E**) and CYP1A2 (**F**) were expressed at similar levels. CYP2E1 protein levels were comparable in whole liver lysates of untreated WT and *Bok^-/-^* mice (**G**,**H**). Results are depicted as a fold change compared to sex-matched WT (**A**–**F**) or as GAPDH normalized values (**H**). Data are represented as mean ± S.D. and are derived from 3 mice per group. *: *p* < 0.05; n.s.: not significant.

The metabolism of APAP into the toxic metabolite NAPQI is mediated by CYPs, especially by CYP2E1 and, to a lesser extent, also by other CYPs, such as CYP1A2 [41]. To test whether expression levels of CYPs differ between *Bok^-/-^* and WT mice, we analyzed the expression of CYP1A2 and CYP2E1 mRNA in primary hepatocytes or whole livers isolated from WT and *Bok^-/-^* mice of both sexes, at baseline conditions (Figure 1A–F) and after APAP treatment (Supplementary Figure S1A,B). Of note, the expression of CYP2E1 at the baseline was increased in both primary hepatocytes and whole liver lysates derived from *Bok^-/-^* male mice compared with WT mice (Figure 1A,C). The expression of CYP1A2 was also increased; however, the difference did not reach statistical significance (Figure 1B,D). On the other hand, no difference in respective CYP expression levels was seen in liver lysates from *Bok^-/-^* and WT female mice (Figure 1E,F). Despite the difference seen at the mRNA level, CYP2E1 protein levels in untreated livers were comparable between the genotypes (Figure 1G,H). After APAP treatment, the expression levels of CYP1A2 and CYP2E1 transcripts did not differ significantly between the two genotypes of either sex. However, CYP2E1, but not CYP1A2, expression decreased in males, while no changes in females were detected (Supplementary Figure S1A,B). CYP2E1 protein levels after APAP treatment were comparable between the genotypes (Supplementary Figure S1C–E).

### 2.2. p53 Expression Correlates with PUMA Expression but Not with the Severity of Liver Damage

**Figure 2:**
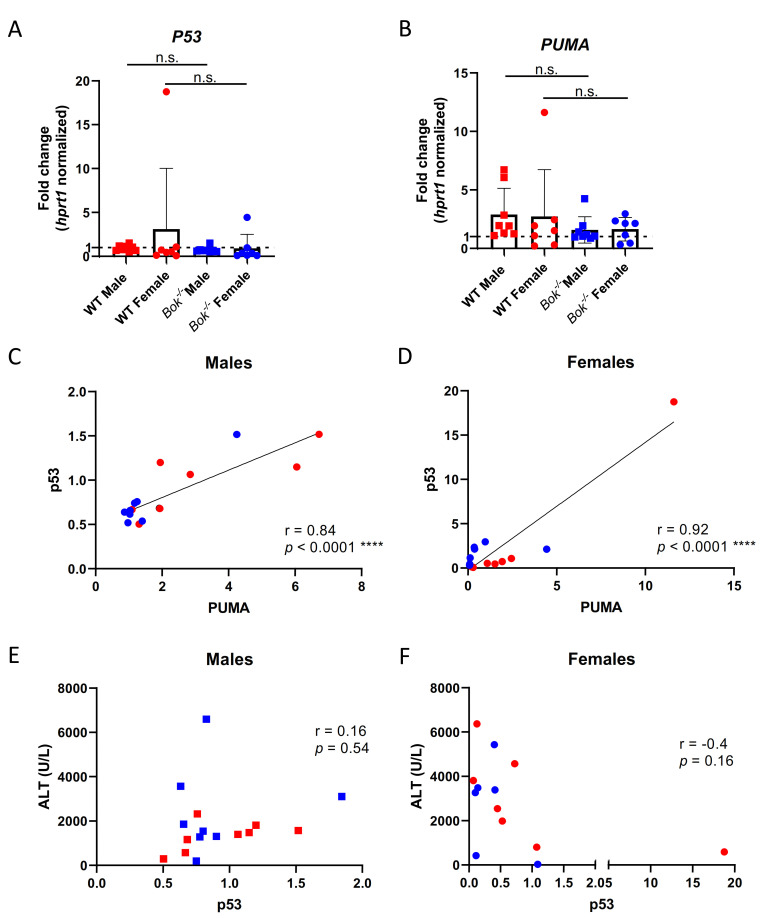
P53 expression correlates with PUMA expression but not with the severity of liver damage. qPCR analysis showed that livers from WT and *Bok^-/-^* mice expressed p53 at similar levels (**A**) and that PUMA was induced in WT and *Bok^-/-^* mice after APAP with higher induction observed in WT mice (**B**). Expression of PUMA and p53 correlated in males (**C**) and females (**D**). P53 expression did not correlate with serum ALT levels in males (**E**) nor in females (**F**). qPCR results are represented as a fold change compared to sex- and genotype matched PBS controls (**A**,**B**). Data are represented as mean ± S.D. and are derived from 8 to 9 mice per group. ****: *p* < 0.0001; n.s.: not significant.

P53 has been described to play a protective and attenuative role in APAP-induced hepatic injury, yet the underlying mechanism is still obscure [42,43,44]. Since we previously found that BOK-deficient cells (HCT116 and HepG2) and liver lysates derived from *Bok^-/-^* mice show a higher baseline expression of p53 at both the transcriptional and protein levels [40,45], we tested p53 expression in the liver after APAP treatment by RT-qPCR. As shown in Figure 2A, similar expression levels of p53 were detected in both sexes of WT and *Bok^/-^* mice. The BH3 only protein and p53 downstream target PUMA has been reported to contribute to APAP-induced hepatotoxicity [21]. Indeed, our data showed that APAP induces the expression of PUMA in WT and in *Bok^-/-^* mice (Figure 2B). Furthermore, p53 expression levels correlated linearly with those of PUMA in both males and females (Figure 2C,D), but not with the severity of liver injury as assessed by the levels of ALT in the serum (Figure 2E,F).

### 2.3. APAP Induces ER Stress and Activates the Unfolded Protein Response

**Figure 3:**
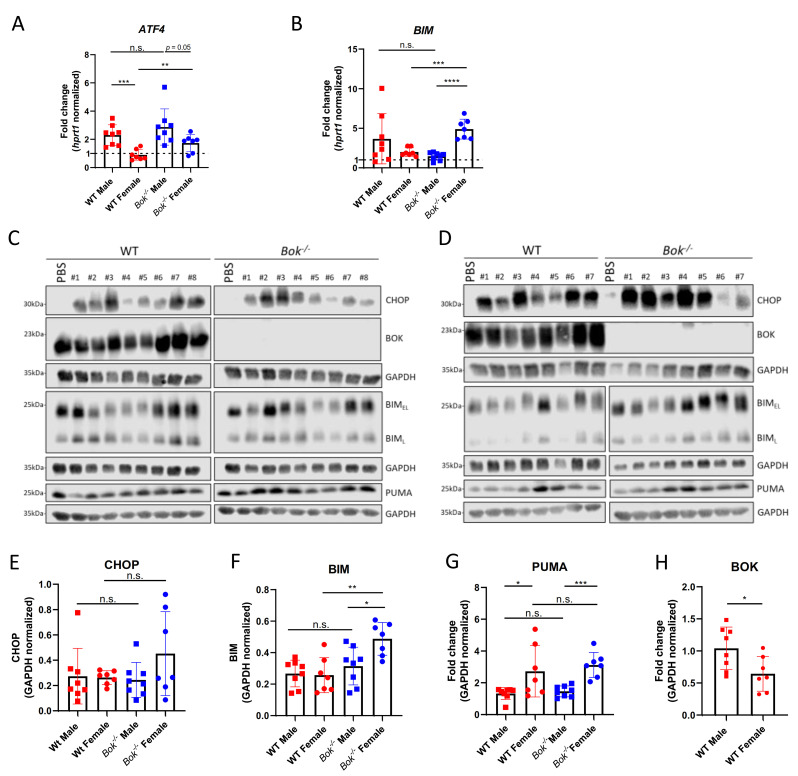
Acetaminophen (APAP) induces ER stress signaling. Transcriptional induction of ATF4 in response to APAP was increased in males compared to females (**A**) and increased in *Bok^-/-^* females compared to WT females (**A**). In all mice, BIM was induced transcriptionally with significantly increased induction in *Bok^-/-^* females compared with *Bok^-/-^* males and WT females (**B**). A Western blot of total liver lysates from males (**C**) and females (**D**) showed similar protein expression levels of BIM and CHOP in WT and *Bok^-/-^* mice, except for higher BIM expression levels in *Bok^-/-^* females (**C**–**F**). PUMA protein was more strongly induced by APAP in females than in males with similar expression levels between genotypes (**C**,**D**,**G**). BCL-2-related ovarian killer (BOK) was downregulated in WT females (**D**,**H**) but not in WT males (**C**,**H**). Results are depicted as a fold change compared to sex- and genotype matched PBS controls (**A**,**B**,**G**,**H**) or as GAPDH normalized values (**E**,**F**). Data are represented as mean ± S.D. and are derived from 8 to 9 mice per group. *: *p* < 0.05; **: *p* < 0.01; ***: *p* < 0.001; ****: *p* < 0.0001; n.s.: not significant.

Several studies reported APAP overdose to induce ER stress [23,24]. Furthermore, there is increasing evidence pointing towards a role for BOK in modulating the unfolded protein response (UPR) [36,39]. Thus, we hypothesized that the activation of the UPR via APAP-triggered ER stress could be impaired in *Bok^-/-^* mice. Therefore, we investigated the expression levels of several proteins implicated in ER stress-induced UPR pathways, among them ATF4, CHOP, BIM and PUMA, in livers of WT and *Bok^-/-^* mice. qRT-PCR analysis revealed that APAP treatment results in an elevation of ATF4 levels in livers of WT and *Bok^-/-^* mice in a sex-specific manner (Figure 3A). In fact, the increase of ATF4 in WT males was comparable to that in *Bok^-/-^* males, but substantially stronger than in females. Interestingly, in the latter, ATF4 induction was more pronounced in *Bok^-/-^* mice than in the WT controls (Figure 3A). This increase in ATF4 in treated *Bok^-/-^* females was associated with increased levels of CHOP and BIM, whereas respective expression levels in males were comparable between the two genotypes (Figure 3B–F). PUMA protein levels were increased after 5 h of APAP treatment compared with PBS controls, and the increase was stronger in females than in males; however, no differences were observed between the genotypes of either sex (Figure 3G) In WT mice, APAP treatment resulted in a 40% downregulation of BOK protein in female but not in male mice at the 5 h timepoint (Figure 3H). 

**Figure 4:**
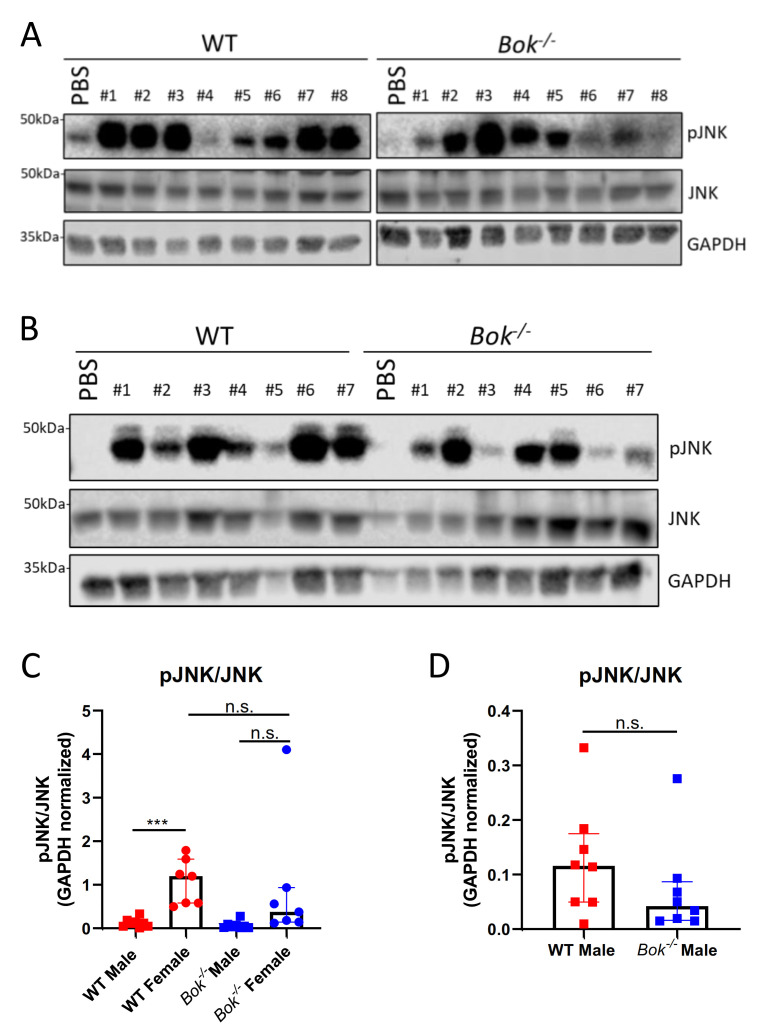
Activation of JNK after APAP treatment. The JNK pathway was activated in livers of males (**A**) and females (**B**) of both genotypes after 5 h of APAP treatment. pJNK was significantly increased in WT females compared with WT males and reduced in both *Bok^-/-^* females (**C**) and males (**D**). (**D**) shows data of male mice from (**C**) as close-up. Data are represented as mean ± S.D. and are derived from 7 to 8 mice per group. ***: *p* < 0.001; n.s.: not significant.

**Figure 5:**
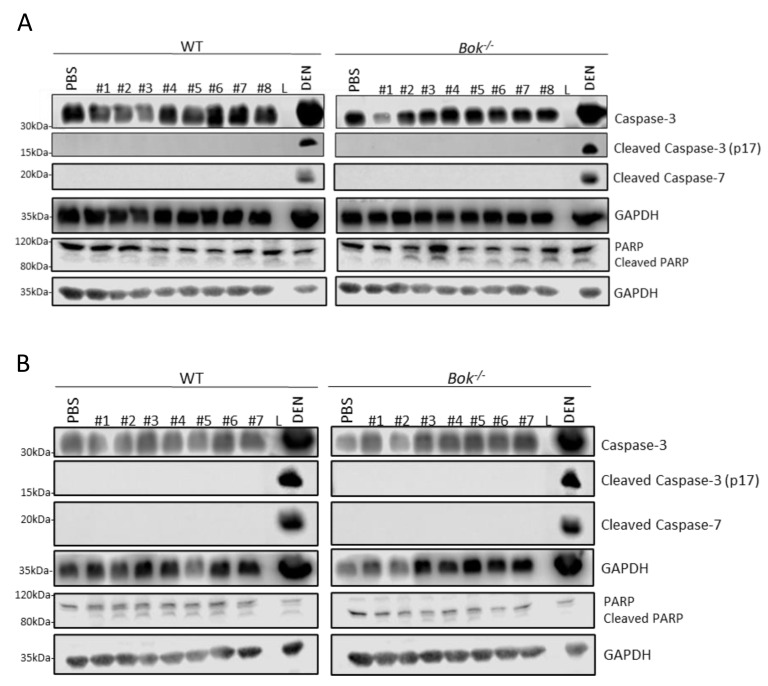
5 h APAP treated livers do not display active effector caspases while PARP cleavage is detectable. No caspase-3 or -7 cleavage was detected in the total liver lysates of males (**A**) and females (**B**) while low degree PARP cleavage was seen after 5 h of APAP treatment. Liver lysate from DEN-treated mice was used as positive control for apoptosis induction [40]. Data are derived from 8 to 9 mice per group.

The JNK signalling pathway was activated in WT and *Bok^-/-^* mice of both sexes. JNK activation was significantly increased in WT females compared to WT males and a similar, although less pronounced, sex-specific effect was seen in mice lacking BOK (Figure 4). In both sexes, a trend of decreased pJNK activation in *Bok^-/-^* mice compared with their WT counterparts was observed (Figure 4). Prolonged activation of the UPR eventually induces apoptosis [46]. However, although our results showed an induction or activation of ER stress-activated proteins, such as JNK, CHOP, BIM and PUMA, all of which have the potential to induce apoptosis, we failed to detect effector caspase-3 or -7 activation at the endpoint of 5 h of APAP treatment, whereas some degree of PARP cleavage was seen (Figure 5). Taken together, we conclude that despite signs of the initiation of classical ER stress-driven apoptotic pathways, effector caspases may have been activated below the detection level, or only during an early phase of APAP overdosing. Alternatively, PARP might have been cleaved by other proteases activated during the course of liver necrosis.

### 2.4. Lack of BOK Slightly Reduces Liver Necrosis after APAP Overdose in a Sex-Dependent Manner

**Figure 6:**
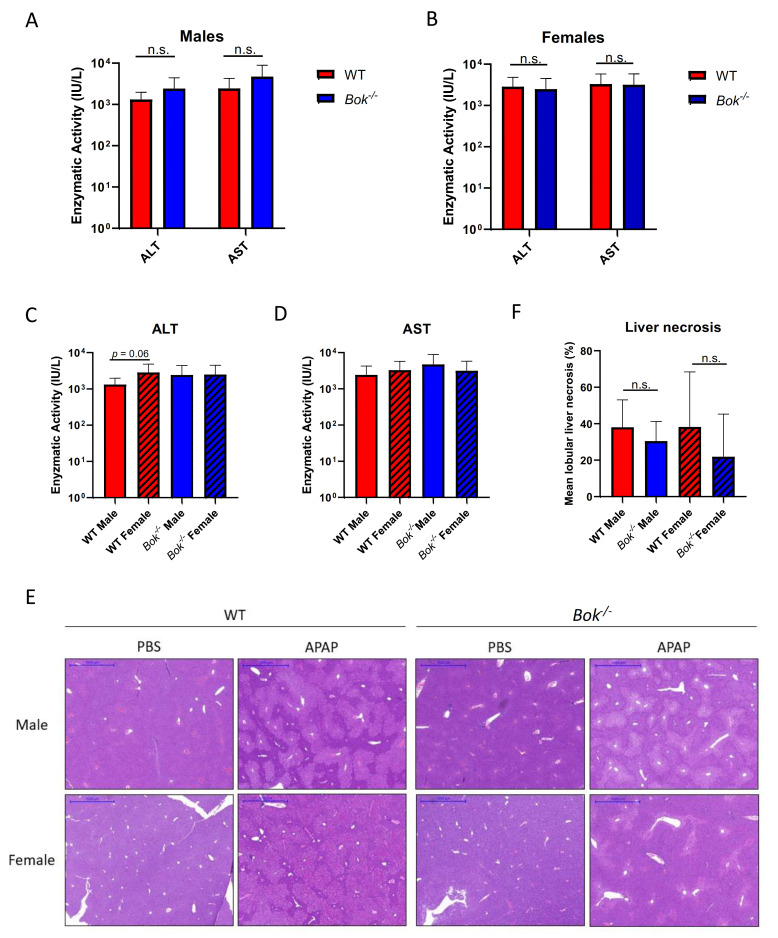
The impact of BOK on the severity of liver damage is moderate and sex dependent. ALT and AST levels were similar in WT and *Bok^-/-^* males (**A**) and females (**B**) after 5 h APAP. ALT and AST levels did not significantly differ between males and females (**C**,**D**). Histologically, centrilobular necrosis was detected in WT and *Bok^-/-^* mice. Haematoxylin and eosin stain (**E**). Scale bars = 1 mm. The extent of centrilobular necrosis (indicated as a mean percentage of affected centrilobular-portal vein distance) was higher in WT mice compared to *Bok^-/-^* mice, with the lowest values in *Bok^-/-^* females (**F**). Data are represented as mean ± S.D. and are derived from 8 mice per group. n.s.: not significant.

The effect of APAP overdosing on inducing liver necrosis was assessed histologically and by quantifying the serum levels of the hepatocellular enzymes ALT and AST. The data showed that when grouping mice of the same sex together, ALT and AST levels were comparable in APAP-treated *Bok^-/-^* and WT mice (Figure 6A,B). However, when comparing male and female mice within a same genotype, slightly increased ALT levels were seen in WT females compared to males (Figure 6C,D). This difference was not seen in *Bok^-/-^* mice (Figure 6C), nor were there differences in AST levels between sex or genotypes (Figure 6D). Histopathological analysis revealed that centrilobular necrosis was found in 100% of WT and *Bok^-/-^* males, whereas in females, necrosis was present in the majority but not all of the WT (75%) and *Bok^-/-^* (62.5%) mice (Table 1). The necrosis was confluent, i.e., bridging, in all animals except one female *Bok^-/-^* animal. The mean extent of lobular necrosis indicated in the percentage of affected centrilobular-portal vein distance was generally higher in WT mice compared with *Bok^-/-^* mice (Table 1). Furthermore, lobular necrosis in *Bok^-/-^* was less extensive in females than in males (21.9% vs. 30.5%) (Figure 6E and Table 1). The hepatic necrosis was haemorrhagic, i.e., admixed with extravasated erythrocytes, in a significant number (50%) of female WT mice (Table 1). This feature was rare or absent in males and female *Bok^-/-^* mice. Altogether, the data indicate that the protective impact upon the loss of BOK on the severity of liver damage is minor, based on histology and ALT/AST levels; however, it becomes more evident when comparing the extent of microscopic lobular necrosis. Of note, these effects in *Bok^-/-^* mice are more strongly pronounced in females, supporting the notion of sex-specific effects in the APAP intoxication model.

## 3. Discussion

APAP intoxication is one of the most common causes for severe liver injury and failure and for subsequent liver transplantation [3]. Although APAP is an over-the-counter drug and one of the most commonly used antipyretic and analgesic drugs in the world, its exact mode of action is unknown. Even less is known about its toxic effect on hepatocytes and the main mechanisms leading to massive cellular damage after ingesting a single overdose (>150 mg/kg or ~7.5 g in adults) or after repetitive intake of APAP or APAP-containing drugs within a short period of time (>200 mg/kg or 10 g within 24 h) [47]. Yet, it is well established that the formation of the highly reactive by-product NAPQI and its binding to cellular, preferentially mitochondrial proteins initiates cellular stress pathways, eventually resulting in hepatocellular necrosis [2,4,6,7].

One of these cellular stress responses is the UPR following ER stress [23,24]. BOK is predominantly localized at the ER and ER-associated membranes and has already been assigned UPR-modulating functions [37,39]. Along these lines, BOK has been shown to promote cell death in a chemical-induced liver injury model [40]. BOK-deficient cells were, on the other hand, reported to be more susceptible to Brefeldin A-induced cell death, pointing towards a UPR modulating function of BOK that can enhance either prosurvival or proapoptotic pathways in a context-dependent manner [37]. We thus hypothesized that after an APAP challenge, *Bok^-/-^* mice would present with a defective ER stress translating either into increased or decreased liver damage. While we did indeed observe slight alterations in the upstream events of ER stress-triggered UPR responses in *Bok^-/-^* mice, this eventually did not result in major differences in APAP-induced liver necrosis compared with WT controls. Nevertheless, our study revealed sex-relevant differences in the activation of ER stress pathways. While the activation of ATF4, a downstream target of PERK, was increased in males, females showed higher expression of pJNK, which is involved in the IRE-1α pathway. Interestingly, it has been shown that estradiol can induce JNK-dependent apoptosis [48,49], suggesting that the activation of the JNK pathway may be sex-dependent with increased activation in females compared to males. Although BOK is known to be important for the regulation of the PERK > ATF4 arm, along with CHOP and BIM [39,50], we did not detect significant differences in the expression levels of any of these members in *Bok^-/-^* males compared to WT males. Whereas we found a correlation between p53 and PUMA transcripts (Figure 2C,D), a direct role of p53 in inducing PUMA in the APAP model is not clear. Actually, Chen et al. proposed a RIPK1 and JNK-dependent, but p53 independent, transcriptional activation of PUMA upon APAP overdosing [21]. On the other hand, the JNK activation in *Bok^-/-^* females was reduced as compared with WT mice, while ATF4 induction, BIM and CHOP expression levels were increased in *Bok^-/-^* females. Since JNK activation was also reduced in *Bok^-/-^* males, we propose that BOK is important for the regulation of the IRE-1α pathway, along with JNK, rather than the PERK > ATF4 arm along with CHOP. Furthermore, the increase of ATF4, CHOP and BIM detected in *Bok^-/-^* compared to WT females can be proposed as an alternative ER stress pathway activation in *Bok^-/-^* females, since the lack of BOK is associated with a reduction in the activation of the IRE-1α arm. The observation that BOK is substantially downregulated in females but not in males supports a sex-dependent role of BOK in the activation of ER stress pathways and for the development of hepatic injury.

It is still unclear and controversially discussed how important apoptosis is for the development of hepatic injury after APAP overdose (reviewed in [14]). Despite the induction of the two pro-apoptotic BH3-only proteins BIM and PUMA in response to APAP, the development of liver damage implicates ER stress likely as a late event [51] in APAP intoxication. In fact, our findings support this view since the expression levels of pJNK and CHOP are still rather low after 3 h intoxication (Supplementary Figure S2).

Another unexpected and sex-related finding was the increased expression levels of CYP2E1 mRNA in livers from *Bok^-/-^* males compared to WT males, which was not seen in females. Increased expression levels of CYPs, induced by e.g., alcohol intake, typically correlate with an increased conversion of APAP into NAPQI and, hence, increased liver damage would be expected for *Bok^-/-^* males [41]. However, CYP2E1 increase was modest and no difference was seen between the genotypes at the protein level. We conclude that the slightly less extensive centrilobular necrosis found in *Bok^-/-^* mice compared with their WT counterparts cannot be explained by changes in CYP2E1 levels. Importantly, the difference in centrilobular necrosis between WT and *Bok^-/-^* mice was higher in females than in males. Thus, BOK seems to be more important for the development of liver damage in females than in males. It has previously been shown that APAP-induced liver damage is sex-specific in mice and that females have a lower susceptibility to APAP hepatotoxicity compared to males [8,9,52]. Du et al. reported a strong protection of C57BL/6 females compared with males in the APAP model. We did not see such a clear difference while using a higher dose of APAP. Of note, in the Du et al. study, the cytosolic pJNK/JNK ratio in females dropped only at a timepoint later than our endpoint of 5 h [8]. However, protection in females is supported by our findings, in which liver necrosis was present in 100% of the males but only 69% (11/16 mice) of the females. This phenomenon may be explained by the finding that females are able to restore GSH faster and thus scavenge more of the toxic NAPQI than males despite similar kinetics of GSH depletion after APAP overdose [8]. In line with these observations, we found total and reduced GSH to be increased in females compared to males; however, no significant difference between WT and *Bok^-/-^* mice was observed (Supplementary Figure S3). This finding suggests that there is no major difference in the metabolism of APAP between WT and *Bok^-/-^* mice.

In conclusion, our data support a role of BOK in the regulation of the UPR after APAP intoxication, especially by modulating the IRE1α > JNK arm that follows a sex-relevant activation pattern reflected by a substantial increase in its activation in female as compared to male mice. However, since apoptosis may not play a major role in the development of necrotic liver damage in this acute APAP overdosing model, the contribution of BOK in regulating the severity of liver damage is more minor after 5 h of intoxication. This view is further supported by our findings that loss of BOK does not alter the sensitivity of mouse and human hepatocellular cell lines towards various doses of APAP in vitro (Supplementary Figure S4). However, all cell models tested except for Tib-75 were rather insensitive to APAP-induced killing, requiring high concentrations to see a decrease in viability (Supplementary Figure S4).

In summary, we show that BOK modulates upstream events linked to ER stress in response to APAP intoxication, and that the impact of BOK on hepatic injury following APAP overdose is only moderate. However, and surprisingly, this difference is sex-dependent, probably due to the differential activation of the UPR in males and females. Since the UPR following ER stress is a rather late event in APAP toxicity, later timepoints could be analysed to identify the involvement of BOK in a subacute intoxication model.

## 4. Materials and Methods

### 4.1. Mice Experiments

Age and sex-matched C57BL/6 WT and *Bok^-/-^* (kindly provided by A. Strasser, WEHI, Parkville, Australia) mice were fasted overnight with free access to water. Following the fasting period, mice were injected with an overdose of APAP (400 mg/kg) dissolved in warm PBS. Control animals were injected with the respective volume of warm PBS. After 5 h, mice were sacrificed, blood was taken by retro-orbital bleeding and livers were collected. The left lobe of the liver of each mouse was fixed in 4% PFA for histology while the others were snap frozen and used later for protein and RNA expression analyses. All animal experiments were approved by the Animal Experimentation Review Board of the Canton of Bern, Switzerland (BE43/19).

### 4.2. Cell Culture

IHH (kindly provided by T. Brunner, University of Konstanz, Konstanz, Germany) were cultured in IMDM (Sigma Aldrich I3390, Buchs, Switzerland), supplemented with 10% FCS, 10^5^ IU/L penicillin-G and 100 mg/L streptomycin and GlutaMAX™ Supplement (Thermo Fisher Scientific, Basel, CH). BNL 1ME A.7R.1 (ATCC^®^ TIB-75™) cells (kind gift of N. Corazza, Bern, Switzerland) were cultured in DMEM (D0819, Sigma-Aldrich, Buchs, Switzerland), supplemented with 10% FCS, 10^5^ IU/L penicillin-G and 100 mg/L streptomycin and 1 mM sodium pyruvate solution (S8636, Sigma Aldrich, Buchs, Switzerland). HepaRG cells (kindly provided by M. Haschke, Bern, Switzerland) were cultured in William’s E medium (Thermo Fisher Scientific, Basel, Switzerland), supplemented with 10% FCS, 10^5^ IU/L penicillin-G and 100 mg/L streptomycin, GlutaMAX™ Supplement, 5 µg/mL bovine insulin (I0516) and 50 µM hydrocortisone hemisuccinate (H2270, Sigma-Aldrich, Buchs, Switzerland).

Primary hepatocytes were isolated as described elsewhere [37] and cultured in collagen-coated flasks in William’s medium E supplemented with 10% FCS, 100 nM dexamethasone (D4902, Sigma-Aldrich, Buchs, Switzerland), GlutaMAX™ Supplement and 10^5^ IU/L penicillin-G and 100 mg/L streptomycin. All cells were kept in a humidified incubator at 37 °C and 5% CO_2_.

### 4.3. CRISPR/Cas9 Gene Editing

The genomic region of BOK was disrupted using the CRISPR/Cas9 technology, as previously described [40]. Two different guide RNAs were used to target human and mouse BOK, respectively. The following primer pair was used to create the gRNA targeting human BOK: F: 5′-caccgGTCTGTGGGCGAGCGGTCAA-3′; R: 5′-aaacTTGACCGCTCGCCCACAGACc-3′. For mouse BOK the primer pair was F: 5′-caccgGTCTGTGGGCGAGCGATCAA-3′; R: 5′-aaacTTGATCGCTCGCCCACA GACc-3′.

### 4.4. Cell Viability Measurements by MTT and Flow Cytometry

HepaRG, IHH and Tib-75 cells were seeded in 96-well flat-bottom plates. Primary hepatocytes were seeded in collagen-coated 96-well flat-bottom plates. After overnight adherence, cells were treated with the indicated concentrations of APAP (Sigma-Aldrich A5000, Buchs, Switzerland) for 24 h. Cell viability of HepaRG, Tib-75 and primary hepatocytes was assessed by 3-(4,5-Dimethyl-2-thiazolyl)-2,5-diphenyl-2H- tetrazoliumbromid (MTT) (Sigma-Aldrich M2128, Buchs, Switzerland). In brief, medium was removed and cells were washed once with PBS. 100 µL MTT solution (5 mg/mL) was added to the cells and incubated for 30 min at 37 °C. After incubation, MTT solution was aspirated and 100 µL DMSO was added into each well. Absorbance was read at 562 nm and cell viability was normalised to untreated control.

For flow cytometric analysis, cells were washed with FACS buffer (150 mM NaCl, 4 mM KCl, 2.5 mM CaCl2, 1 mM MgSO4, 15 mM HEPES pH 7.2, 2% FCS and 10 mM NaN3) and incubated with FITC–Annexin V diluted in FACS buffer. After 20 min of incubation on ice in the dark, cells were washed with FACS buffer and resuspended in 100 µL FACS buffer containing 2 µg/mL propidium iodide. Cells were examined by a BD FACSLyric™ Research System (BD Biosciences, San Jose, CA, USA). Double negative cells were considered as viable cells.

### 4.5. Immunoblotting

Frozen liver pieces were homogenized using the Mikro-Dismembrator S (Sartorius Group) and lysed in RIPA buffer (50 mM Tris-HCl pH 8.0, 150 mM NaCl, 1% Triton X100, 0.5% Na-deoxycholate, 0.1% SDS) containing cOmplete™ Protease Inhibitor Cocktail (Roche), pepstatin (1 mg/mL) and phosphatase inhibitors (2 mM sodium orthovanadate and 50 mM sodium fluoride). Samples were boiled in Lämmli buffer containing 100 mM DTT, separated on 12.5% SDS-PAGE gels and transferred to Immobilon^®^-FL PVDF membranes (Merck). Membranes were probed with mouse anti-CHOP (B-3) (Santa Cruz Biotechnology, sc-7351), rabbit anti-CYP2E1 (Merck # HPA009128), mouse anti-pJNK (G9) (#9255, Cell Signaling Technology, Danvers, MA, USA), rabbit anti-JNK (Cell Signaling Technology #9252), rabbit anti-GAPDH (14C10) (Cell Signaling Technology #2118), rabbit anti-BIM (C34C5) (Cell Signaling Technology #2933), rabbit anti-PUMA (Cell Signaling Technology #7467), rabbit anti-cleaved caspase-3 (5A1E) (Cell Signaling Technology #9664), rabbit anti-caspase-3 (Cell Signaling Technology #9662), rabbit anti-cleaved Caspase-7 (Cell Signaling Technology #9491), mouse anti-PARP (clone C2-10; BD Biosciences, San Jose, CA, USA) and rabbit anti-BOK (clone BOK-1-5) [39]. Fluorochrome-conjugated secondary antibodies, IRDye^®^ 800CW and IRDye^®^ 600RD (LI-COR Biotechnology, Bad Homburg, DE), or horseradish-peroxidase conjugated secondary antibodies (Jackson ImmunoResearch Europe Ltd., Ely, UK) were used. For the latter, the membrane was additionally incubated in Immobilon Forte Western HRP substrate (Merck). Signals were detected by the Odyssey^®^ Fc Imaging System (LI-COR Biosciences) and data was quantified using Image Studio Lite 5.2 software (LI-COR Biosciences).

### 4.6. Total and Reduced Glutathione (GSH) Measurement

20 mg liver tissue of each mouse was resuspended in ice-cold lysis buffer (0.5% NP40 in PBS). Samples were deproteinized using trichloroacetic acid (TCA) and neutralized using sodium hydrogen carbonate (NaHCO3). Total and reduced GSH levels were measured using the GSH/GSSG Ratio Detection Assay Kit II, according to the manufacturer’s instructions (ab205811, abcam, Cambridge, MA, USA).

### 4.7. RNA Extraction and Quantitative RT-PCR (qPCR)

Total RNA of liver tissue was isolated using the SV Total RNA Isolation System (Promega). One µg RNA was reverse transcribed using M-MLV Reverse Transcriptase following the manufacturer’s instructions (Promega). Real-time PCR for mouse *Atf4* (F: 5′-TCGATGCTCTGTTTCGAATG-3′; R: 5′-GGCAACCTGGTC GACTTTTAT-3′), mouse *Bim* (F: 5′-GAGTTGTGACAAGTCAACACAAACC-3′; R: 5′-GAAGATAAAGCGTAACAGTTGTAAGATAACC-3′), mouse *Puma* (F: 5′-ATGCCTGCCTCACCTTCATCT3′; R: 5′-AGCACAGGATTCACAGTCTGGA-3′), mouse *Tp53* (F: 5′-CACGTACTCTCCTCCCCTCAAT-3′; R: 5′-AACTGCACA GGGCACGTCTT-3′), mouse *Bok* (F: 5′-TTCTCAGCAGGTATCACATGG-3′; R: 5′-TAGCCAAGGTCTTGCGTACA-3′), mouse *Cyp1a2* (F: 5′-CCAGGTGGTGGAAT CGGTG-3′; R: 5′-TCTTAAACCTCTTGAGGGCCG-3′) and mouse *Cyp2e1* (F: 5′-CATCACCGTTGCCTTGCTTG-3′; R: 5′GCCAACTTGGTTAAAGACTTGGG-3′) was performed in a CFX Connect Real-Time PCR Detection System (Bio-Rad) using HOT FIREPol ‘EvaGreen’ qPCR Mix Plus (Solis Biodyne, Tartu, EST). Mouse *Hprt1* (F: TGGATACAGGCCAGACTTTGTT; R: CAGATTCAACTTGCGCTCATC) and/or mouse *β-actin* (F: GCTTCTTTGCAGCTCCTTCGT; R: CGTCATCCATGG CGAACTG) were used as reference genes.

### 4.8. Histology

Freshly isolated liver lobes were fixed in 4% paraformaldehyde overnight, embedded in paraffin and cut in 4 µM sections. The sections were stained with hematoxylin and eosin. Histopathological analysis was performed blinded by a veterinary pathologist (SdB). The extent of lobular liver necrosis was calculated by measuring the percentage of affected centrilobular-portal vein distance. For this purpose, five randomly selected different measurements were taken of each liver tissue fragment and corresponding mean values were calculated for each liver tissue fragment and each animal.

### 4.9. Statistical Analysis

Statistical differences were analysed using the unpaired Student’s t-test. *p*-values of <0.05 were considered significant (*: *p* < 0.05; **: *p* < 0.01; ***: *p* < 0.001; ****: *p* < 0.0001; n.s.: not significant). Correlation analyses were performed using the Pearson correlation coefficient. Graphs were created using Prism 7.0 (GraphPad, San Diego, CA, USA).

## Figures and Tables

**Table 1:** Histopathological assessment of liver damage in APAP treated WT and *Bok^-/-^* mice.

Group	Presence of Centrilobular Necrosis	Extent of Lobular Necrosis (%) Mean (Range, SD)	Mean Lobular Necrosis (%) Excluding Cases with Absent Necrosis	Centrilobular Vacuolar Change	Presence of Bridging Necrosis	Presence of Hemorrhagic Necrosis
WT Male	8/8 (100%)	38.13 (23–60, SD 14.98)	38.13 (SD 14.98)	8/8 (100%)	8/8 (100%)	1/8 (12.5%)
*Bok^-/-^* Male	8/8 (100%)	30.5 (18–45, SD 10.74)	30.5 (SD 10.74)	8/8 (100%)	8/8 (100%)	0/8
WT Female	6/8 (75%)	38.25 (0–73, SD 30.16)	51 (SD 22.20)	7/8 (87.5%)	8/8 (100%)	4/8 (50%)
*Bok^-/-^* Female	5/8 (62.5%)	21.88 (0–71, SD 23.41)	29.17 (SD 22.62)	7/8 (87.5%)	7/8 (87.5%)	1/8 (12.5%)

## Supplementary Materials

The following are available online at https://www.mdpi.com/1422-0067/22/6/3281/s1.

## References

[1] Ghanem C.I., Perez M.J., Manautou J.E., Mottino A.D. (2016). Acetaminophen from liver to brain: New insights into drug pharmacological action and toxicity. Pharmacol. Res..

[2] Ramachandran A., Jaeschke H. (2019). Acetaminophen hepatotoxicity. Semin. Liver Dis..

[3] Jaeschke H. (2015). Acetaminophen: Dose-dependent drug hepatotoxicity and acute liver failure in patients. Dig. Dis..

[4] Ramachandran A., Jaeschke H. (2018). Acetaminophen toxicity: Novel insights into mechanisms and future perspectives. Gene Expr..

[5] Bessems J.G., Vermeulen N.P. (2001). Paracetamol (acetaminophen)-induced toxicity: Molecular and biochemical mechanisms, analogues and protective approaches. Crit. Rev. Toxicol..

[6] Moles A., Torres S., Baulies A., Garcia-Ruiz C., Fernandez-Checa J.C. (2018). Mitochondrial-lysosomal axis in acetaminophen hepatotoxicity. Front. Pharmacol..

[7] Chao X., Wang H., Jaeschke H., Ding W.X. (2018). Role and mechanisms of autophagy in acetaminophen-induced liver injury. Liver Int..

[8] Du K., Williams C.D., McGill M.R., Jaeschke H. (2014). Lower susceptibility of female mice to acetaminophen hepatotoxicity: Role of mitochondrial glutathione, oxidant stress and c-jun n-terminal kinase. Toxicol. Appl. Pharmacol..

[9] Dai G., He L., Chou N., Wan Y.J. (2006). Acetaminophen metabolism does not contribute to gender difference in its hepatotoxicity in mouse. Toxicol. Sci..

[10] Mitchell J.R., Jollow D.J., Potter W.Z., Davis D.C., Gillette J.R., Brodie B.B. (1973). Acetaminophen-induced hepatic necrosis. I. Role of drug metabolism. J. Pharmacol. Exp. Ther..

[11] Yamada N., Karasawa T., Kimura H., Watanabe S., Komada T., Kamata R., Sampilvanjil A., Ito J., Nakagawa K., Kuwata H. (2020). Ferroptosis driven by radical oxidation of n-6 polyunsaturated fatty acids mediates acetaminophen-induced acute liver failure. Cell Death Dis..

[12] Lorincz T., Jemnitz K., Kardon T., Mandl J., Szarka A. (2015). Ferroptosis is involved in acetaminophen induced cell death. Pathol. Oncol. Res..

[13] Jaeschke H., Ramachandran A., Chao X., Ding W.X. (2019). Emerging and established modes of cell death during acetaminophen-induced liver injury. Arch. Toxicol..

[14] Jaeschke H., Duan L., Akakpo J.Y., Farhood A., Ramachandran A. (2018). The role of apoptosis in acetaminophen hepatotoxicity. Food Chem. Toxicol..

[15] Ge Z., Wang C., Zhang J., Li X., Hu J. (2019). Tempol protects against acetaminophen induced acute hepatotoxicity by inhibiting oxidative stress and apoptosis. Front. Physiol..

[16] Kumari A., Kakkar P. (2012). Lupeol protects against acetaminophen-induced oxidative stress and cell death in rat primary hepatocytes. Food Chem. Toxicol..

[17] Ahmad S.T., Arjumand W., Nafees S., Seth A., Ali N., Rashid S., Sultana S. (2012). Hesperidin alleviates acetaminophen induced toxicity in wistar rats by abrogation of oxidative stress, apoptosis and inflammation. Toxicol. Lett..

[18] Adams M.L., Pierce R.H., Vail M.E., White C.C., Tonge R.P., Kavanagh T.J., Fausto N., Nelson S.D., Bruschi S.A. (2001). Enhanced acetaminophen hepatotoxicity in transgenic mice overexpressing bcl-2. Mol. Pharmacol..

[19] Badmann A., Keough A., Kaufmann T., Bouillet P., Brunner T., Corazza N. (2011). Role of trail and the pro-apoptotic bcl-2 homolog bim in acetaminophen-induced liver damage. Cell Death Dis..

[20] Maxa M., Schaeper U., Dames S., Vollmar B., Kuhla A. (2019). Liver-specific bid silencing inhibits apap-induced cell death in mice. Apoptosis.

[21] Chen D., Ni H.M., Wang L., Ma X., Yu J., Ding W.X., Zhang L. (2019). P53 up-regulated modulator of apoptosis induction mediates acetaminophen-induced necrosis and liver injury in mice. Hepatology.

[22] Jaeschke H., Duan L., Nguyen N., Ramachandran A. (2019). Mitochondrial damage and biogenesis in acetaminophen-induced liver injury. Liver Res..

[23] Lorz C., Justo P., Sanz A., Subira D., Egido J., Ortiz A. (2004). Paracetamol-induced renal tubular injury: A role for er stress. J. Am. Soc. Nephrol..

[24] Nagy G., Kardon T., Wunderlich L., Szarka A., Kiss A., Schaff Z., Banhegyi G., Mandl J. (2007). Acetaminophen induces er dependent signaling in mouse liver. Arch. Biochem. Biophys..

[25] Puthalakath H., O’Reilly L.A., Gunn P., Lee L., Kelly P.N., Huntington N.D., Hughes P.D., Michalak E.M., McKimm-Breschkin J., Motoyama N. (2007). Er stress triggers apoptosis by activating bh3-only protein bim. Cell.

[26] Torres S., Baulies A., Insausti-Urkia N., Alarcon-Vila C., Fucho R., Solsona-Vilarrasa E., Nunez S., Robles D., Ribas V., Wakefield L. (2019). Endoplasmic reticulum stress-induced upregulation of stard1 promotes acetaminophen-induced acute liver failure. Gastroenterology.

[27] Lee D.H., Lee B., Park J.S., Lee Y.S., Kim J.H., Cho Y., Jo Y., Kim H.S., Lee Y.H., Nam K.T. (2019). Inactivation of sirtuin2 protects mice from acetaminophen-induced liver injury: Possible involvement of er stress and s6k1 activation. BMB Rep..

[28] Uzi D., Barda L., Scaiewicz V., Mills M., Mueller T., Gonzalez-Rodriguez A., Valverde A.M., Iwawaki T., Nahmias Y., Xavier R. (2013). Chop is a critical regulator of acetaminophen-induced hepatotoxicity. J. Hepatol..

[29] Saveljeva S., Mc Laughlin S.L., Vandenabeele P., Samali A., Bertrand M.J. (2015). Endoplasmic reticulum stress induces ligand-independent tnfr1-mediated necroptosis in l929 cells. Cell Death Dis..

[30] Chavez-Valdez R., Flock D.L., Martin L.J., Northington F.J. (2016). Endoplasmic reticulum pathology and stress response in neurons precede programmed necrosis after neonatal hypoxia-ischemia. Int. J. Dev. Neurosci..

[31] Zhong W., Wang X., Rao Z., Pan X., Sun Y., Jiang T., Wang P., Zhou H., Wang X. (2020). Aging aggravated liver ischemia and reperfusion injury by promoting hepatocyte necroptosis in an endoplasmic reticulum stress-dependent manner. Ann. Transl. Med..

[32] Ke F.F.S., Vanyai H.K., Cowan A.D., Delbridge A.R.D., Whitehead L., Grabow S., Czabotar P.E., Voss A.K., Strasser A. (2018). Embryogenesis and adult life in the absence of intrinsic apoptosis effectors bax, bak, and bok. Cell.

[33] Moldoveanu T., Zheng J.H. (2018). Metastability, an emerging concept governing bok-mediated apoptosis initiation. Oncotarget.

[34] Einsele-Scholz S., Malmsheimer S., Bertram K., Stehle D., Johanning J., Manz M., Daniel P.T., Gillissen B.F., Schulze-Osthoff K., Essmann F. (2016). Bok is a genuine multi-bh-domain protein that triggers apoptosis in the absence of bax and bak. J. Cell Sci..

[35] Llambi F., Wang Y.M., Victor B., Yang M., Schneider D.M., Gingras S., Parsons M.J., Zheng J.H., Brown S.A., Pelletier S. (2016). Bok is a non-canonical bcl-2 family effector of apoptosis regulated by er-associated degradation. Cell.

[36] Naim S., Kaufmann T. (2020). The multifaceted roles of the bcl-2 family member bok. Front. Cell Devel. Biol..

[37] Echeverry N., Bachmann D., Ke F., Strasser A., Simon H.U., Kaufmann T. (2013). Intracellular localization of the bcl-2 family member bok and functional implications. Cell Death Differ..

[38] Schulman J.J., Wright F.A., Han X., Zluhan E.J., Szczesniak L.M., Wojcikiewicz R.J. (2016). The stability and expression level of bok are governed by binding to inositol 1,4,5-trisphosphate receptors. J. Biol. Chem..

[39] Carpio M.A., Michaud M., Zhou W., Fisher J.K., Walensky L.D., Katz S.G. (2015). Bcl-2 family member bok promotes apoptosis in response to endoplasmic reticulum stress. Proc. Natl. Acad. Sci. USA.

[40] Rabachini T., Fernandez-Marrero Y., Montani M., Loforese G., Sladky V., He Z., Bachmann D., Wicki S., Villunger A., Stroka D. (2018). Bok promotes chemical-induced hepatocarcinogenesis in mice. Cell Death Differ..

[41] Kalsi S.S., Wood D.M., Waring W.S., Dargan P.I. (2011). Does cytochrome p450 liver isoenzyme induction increase the risk of liver toxicity after paracetamol overdose?. Open Access Emerg. Med..

[42] Huo Y., Yin S., Yan M., Win S., Aung Than T., Aghajan M., Hu H., Kaplowitz N. (2017). Protective role of p53 in acetaminophen hepatotoxicity. Free Radic. Biol. Med..

[43] Borude P., Bhushan B., Gunewardena S., Akakpo J., Jaeschke H., Apte U. (2018). Pleiotropic role of p53 in injury and liver regeneration after acetaminophen overdose. Am. J. Pathol..

[44] Sun J., Wen Y., Zhou Y., Jiang Y., Chen Y., Zhang H., Guan L., Yao X., Huang M., Bi H. (2018). P53 attenuates acetaminophen-induced hepatotoxicity by regulating drug-metabolizing enzymes and transporter expression. Cell Death Dis..

[45] Srivastava R., Cao Z., Nedeva C., Naim S., Bachmann D., Rabachini T., Gangoda L., Shahi S., Glab J., Menassa J. (2019). Bcl-2 family protein bok is a positive regulator of uridine metabolism in mammals. Proc. Natl. Acad. Sci. USA.

[46] Fribley A., Zhang K., Kaufman R.J. (2009). Regulation of apoptosis by the unfolded protein response. Methods Mol. Biol..

[47] Dart R.C., Erdman A.R., Olson K.R., Christianson G., Manoguerra A.S., Chyka P.A., Caravati E.M., Wax P.M., Keyes D.C., Woolf A.D. (2006). Acetaminophen poisoning: An evidence-based consensus guideline for out-of-hospital management. Clin. Toxicol. (Phila).

[48] Altiok N., Ersoz M., Koyuturk M. (2011). Estradiol induces jnk-dependent apoptosis in glioblastoma cells. Oncol. Lett..

[49] Altiok N., Koyuturk M., Altiok S. (2007). Jnk pathway regulates estradiol-induced apoptosis in hormone-dependent human breast cancer cells. Breast Cancer Res. Treat..

[50] Moravcikova E., Krepela E., Donnenberg V.S., Donnenberg A.D., Benkova K., Rabachini T., Fernandez-Marrero Y., Bachmann D., Kaufmann T. (2017). Bok displays cell death-independent tumor suppressor activity in non-small-cell lung carcinoma. Int. J. Cancer.

[51] Foufelle F., Fromenty B. (2016). Role of endoplasmic reticulum stress in drug-induced toxicity. Pharmacol. Res. Perspect..

[52] Mohar I., Stamper B.D., Rademacher P.M., White C.C., Nelson S.D., Kavanagh T.J. (2014). Acetaminophen-induced liver damage in mice is associated with gender-specific adduction of peroxiredoxin-6. Redox Biol..

