# Supplementary material for: Loss of BOK Has a Minor Impact on Acetaminophen Overdose-Induced Liver Damage in Mice
Source: Int J Mol Sci. 2021 Mar 23;22(6):3281. doi: 10.3390/ijms22063281 (PMC8004760; doi:10.3390/ijms22063281)
Supplement: Supplementary file 1 [file ijms-22-03281-s001.pdf]

Loss of BOK has a minor impact on acetaminophen overdose-induced liver damage in mice

Samara Naim[1], Yuniel Fernandez-Marrero[1,2], Simone de Brot[3], Daniel Bachmann[1], and Thomas Kaufmann[1,*]

[1] Institute of Pharmacology, University of Bern, Inselspital, INO-F, 3010 Bern, Switzerland

[2] Current address: Sunnybrook Health Sciences Centre, University of Toronto, 2075 Bayview Ave, Toronto, ON M4N 3M5

[3] COMPATH, Institute of Animal Pathology, University of Bern, Laenggassstrasse 122, CH-3012 Bern

[*] Correspondence: thomas.kaufmann@pki.unibe.ch

Online Supplementary Figures S1 – S4

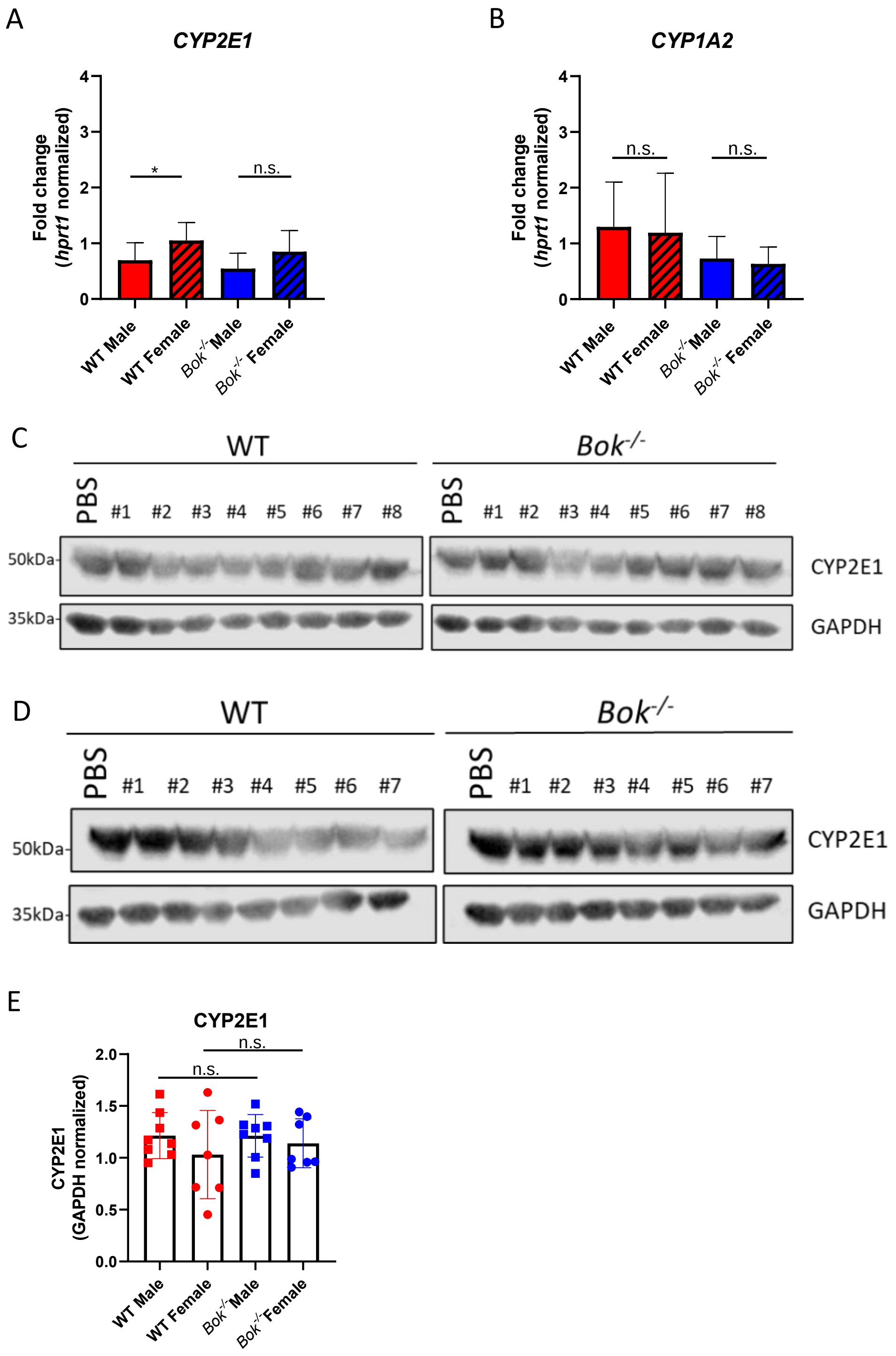

Supplementary Figure S1. CYP expression levels after APAP treatment. After 5h of APAP treatment, no significant differences in mRNA expression levels of CYP2E1 or CYP1A2 were detected between livers from WT and *Bok*$^{-/-}$ mice, except for a slight decrease of CYP2E1 in WT males compared with WT females (A, B). CYP2E1 protein levels did not differ in livers of WT and *Bok*$^{-/-}$ mice after 5h of APAP treatment (C-E). Results are depicted as fold change compared to sex-matched WT (A,B) or presented as GAPDH normalized values (E). Data are represented as mean ± S.D. and are derived from 8 to 9 mice per group.

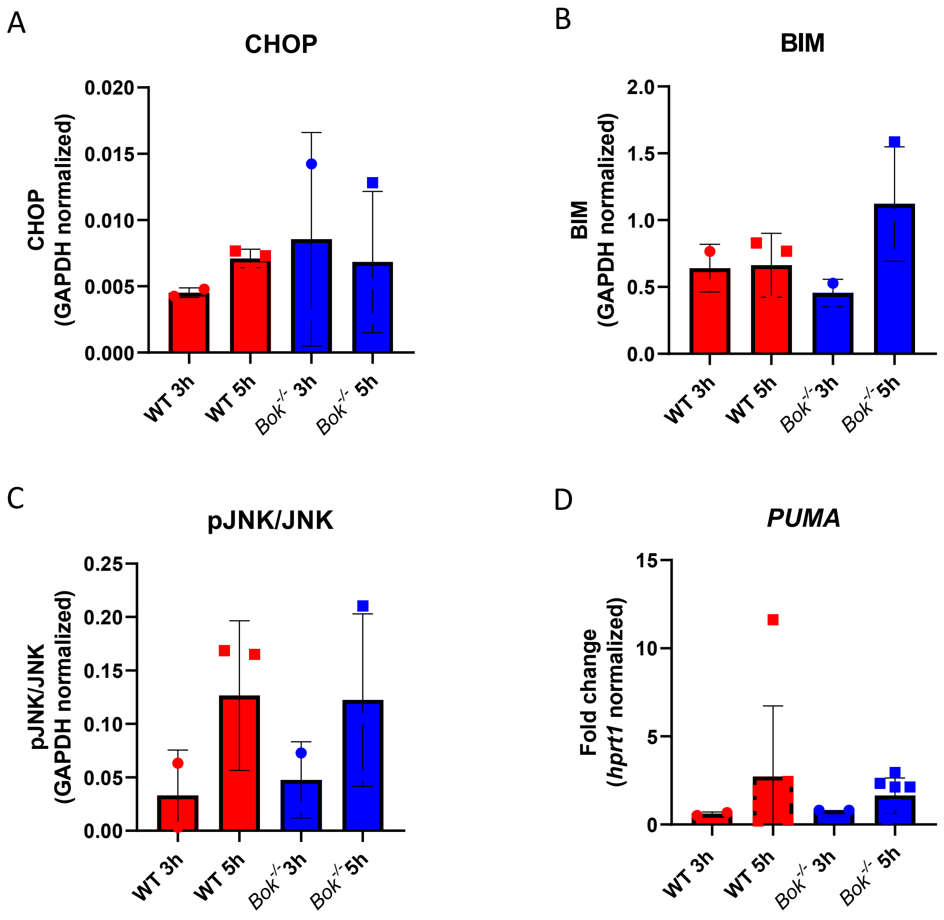

Supplementary Figure S2. ER stress is a late event after APAP overdose. Expression levels of CHOP (A), BIM (B) and pJNK/JNK (C) protein and PUMA mRNA (D) in livers of WT and *Bok*$^{-/-}$ mice after 3h and 5h of APAP treatment. Transcriptional induction of PUMA is represented as fold increase compared to sex-and genotype matched PBS controls (D). Data are represented as mean ± S.D. and are derived from 2 to 3 female mice per group.

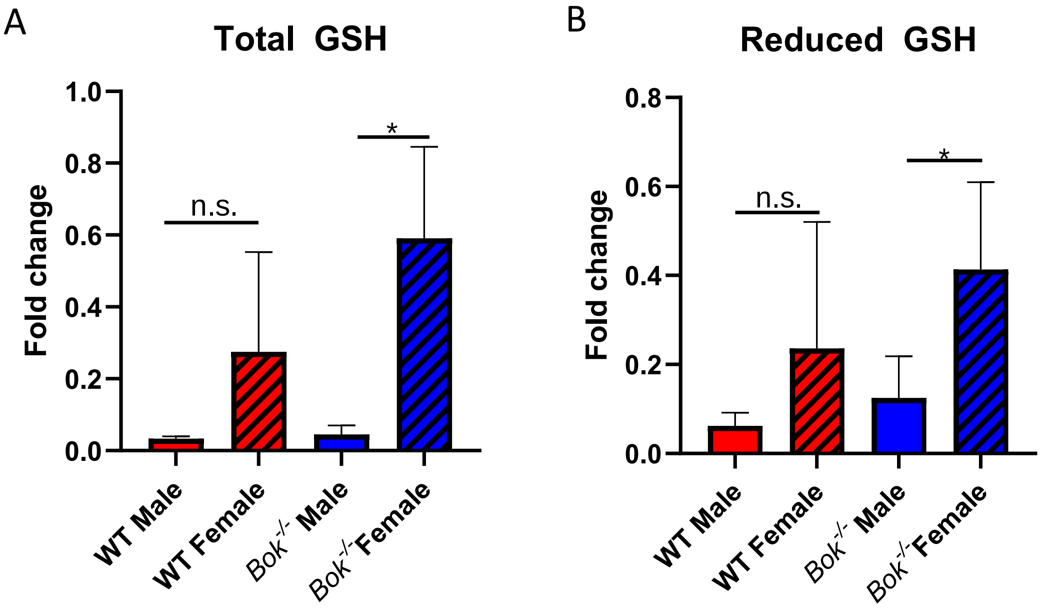

Supplementary Figure S3. Females have higher levels of total and reduced GSH after APAP overdose compared to males. Livers of WT and *Bok*$^{-/-}$ females showed higher levels of total GSH (A) and reduced GSH (B) compared to their male counterparts. Data show fold change compared to respective PBS controls and are represented as mean ± S.D. and are derived from 8 to 9 mice per group.

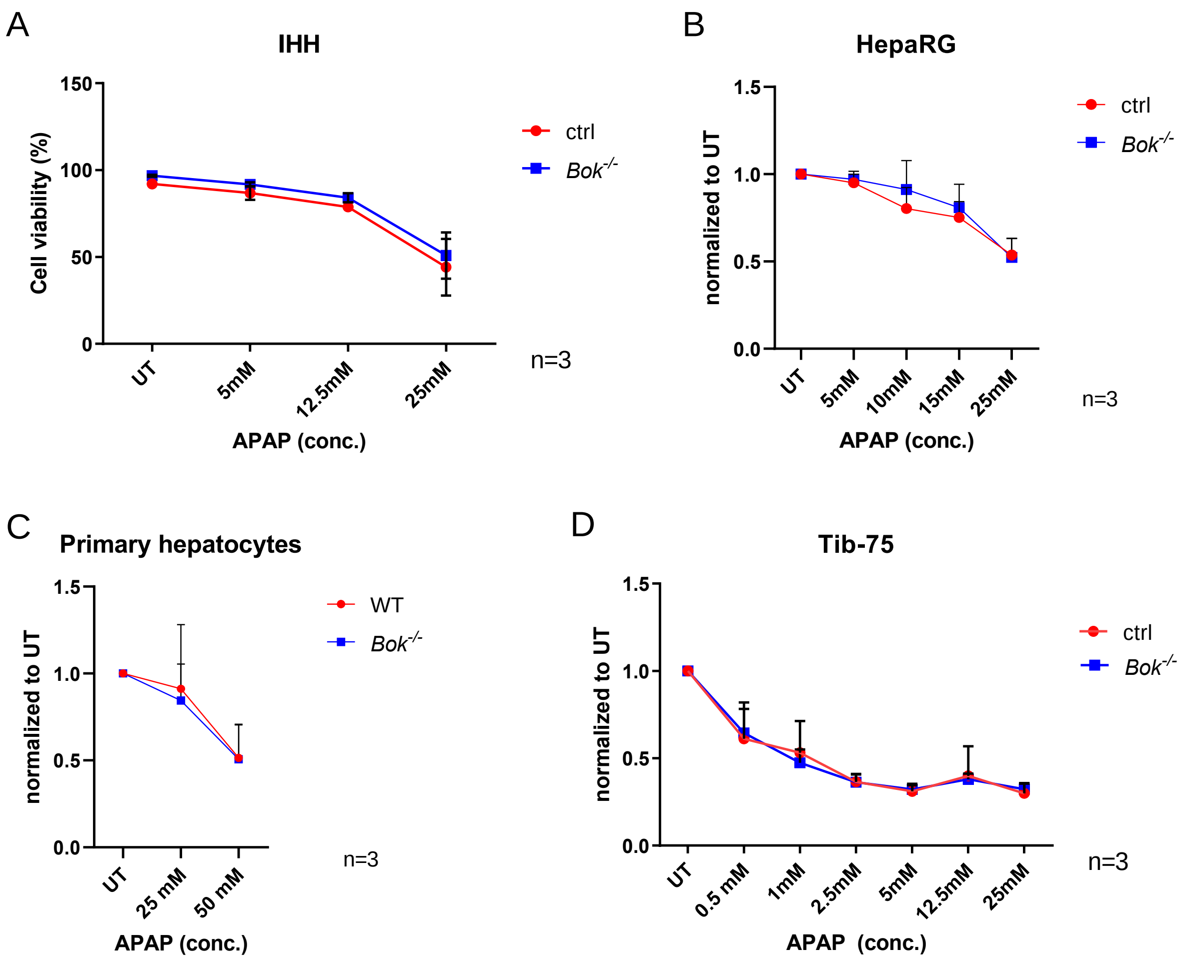

Supplementary Figure S4. *Bok$^{-/-}$* cell lines have the same sensitivity towards various doses of APAP. The lack of BOK in the human cell lines IHH (A) and HepaRG (B), as well as in primary mouse hepatocytes (C) and the murine liver cell line Tib-75 (D) did not change the rate of cell death in response to various doses of APAP. Cell viability of IHH was measured by Annexin/PI staining using flow cytometry and the percentage of Annexin/PI double negative (viable) cells is depicted (A). Viability of HepaRG, mouse primary hepatocytes and Tib-75 was measured by MTT and normalized to untreated controls (B-D). Data are represented as mean ± S.D. and are derived from 3 independent experiments.
